# Increased energy expenditure during posture maintenance and exercise in early Parkinson disease

**DOI:** 10.1002/hsr2.14

**Published:** 2017-08-22

**Authors:** Tal Margaliot Kalifa, Nadav Ziv, Hagai Bergman, Samir Nusair, David Arkadir

**Affiliations:** ^1^ The Hebrew University–Hadassah Medical School Jerusalem Israel; ^2^ Medical Physiology Laboratory The Hebrew University–Hadassah Medical School Jerusalem Israel; ^3^ Department of Medical Neurobiology, The Institute of Medical Research Israel‐Canada (IMRIC), and Edmond and Lily Safra Center for Brain Research (ELSC) The Hebrew University Jerusalem Israel; ^4^ Department of Neurosurgery Hadassah‐Hebrew University Medical Center Jerusalem Israel; ^5^ Institute of Pulmonary Medicine Hadassah‐Hebrew University Medical Center Jerusalem Israel; ^6^ Rokach Center for the Prevention of Lung Diseases, Clalit Health Services, Affiliated to the School of Medicine Hebrew University and Hadassah Jerusalem Israel; ^7^ Department of Neurology Hadassah‐Hebrew University Medical Center Jerusalem Israel

**Keywords:** energy expenditure, exercise, indirect calorimetry, Parkinson disease, weight loss

## Abstract

**Background:**

Evidence for the effects of Parkinson disease on energy expenditure is incomplete and contradictory. A number of studies showed increased resting energy expenditure among patients with Parkinson disease whereas others did not. It was hypothesized that energy expenditure increases during exercise, based on findings in patients with a variable regime of anti‐parkinsonian therapies and at different stages of the disease. However, energy expenditure during posture maintenance has been neglected. To better understand these issues, we studied energy expenditure in a homogenous population of Parkinson patients in an early stage of the disease and different states of activity.

**Methods:**

Oxygen consumption was assessed in a group of 10 males with early Parkinson disease without dopaminergic treatment and controls matched for age and body composition. Oxygen consumption was measured at rest, during trunk unsupported sitting, and during exercise at different intensities (unloaded and loaded cycling).

**Results:**

Resting energy expenditure was similar between groups. Higher energy consumption was observed during maintenance of trunk posture at rest and during light intensity aerobic exercise (P < .05 for all conditions). The increment in energy expenditure associated with increased physical demand tended to be steeper in Parkinson disease.

**Conclusion:**

Resting energy expenditure is normal in Parkinson disease. However, energy expenditure increases during physical activity and even during the maintenance of unsupported posture among patients with Parkinson disease.

## Abstract 2

It has been hypothesized that weight loss in Parkinson disease (PD) is the result of increased resting energy expenditure, yet conclusive evidence for this hypothesis has been lacking. This study demonstrated that increased energy expenditure in PD patients occurs only during posture maintenance and physical activity, not during rest. Evidence for abnormally increased energy expenditure emerged as of the recruitment of axial muscles to support the body trunk in a sitting posture.

## 1 INTRODUCTION

Unexplained loss of weight in the early phases of Parkinson disease (PD) is common.^1^ In cases of normal (or even increased) caloric intake in early PD,^1^, ^2^ the cause of weight loss in this phase of the disease is often assumed to be increased energy expenditure. This hypothesis has been supported by certain studies that have reported increased energy expenditure in PD,^3^, ^4^ but disconfirmed by others.^2^, ^5^ These conflicting findings may be associated with differences in the use of dopaminergic agents, the severity of the disease, body composition, and/or unmatched gender between PD and control groups. Some studies have not made a distinction between resting energy expenditure and activity‐induced energy expenditure, and the experimental procedures were not designed to address these putative differences.

To better understand energy expenditure in PD, we measured oxygen consumption (V̇O_2_) at rest and during moderate aerobic effort under optimally controlled conditions, in a homogeneous group of PD male patients, naive to dopaminergic therapy, in the early phase of the disease.

## 2 METHODS

### 2.1 Participants

Ten male PD patients, aged 42 to 74 years, were recruited through the Hadassah Medical Center's neurology outpatient Clinic for Movement Disorders. Ten non‐parkinsonian and non–blood‐related male individuals served as age‐matched controls. The exclusion criteria included significant cognitive impairment, clinically significant cardiovascular conditions, drug‐induced parkinsonism, suspected Parkinson‐plus syndrome, and the use of medication that is known to modulate metabolic rate such as beta‐blockers or thyroid replacement therapy. The study was approved by the Institutional Review Board committee of Hadassah Medical Center (HMO‐0078‐15) and has therefore been performed in accordance with the ethical standards laid down in the 1964 Declaration of Helsinki and its later amendments. All participants signed an informed consent before enrollment. A physician was present during all exercises.

To reduce the variability among participants (percentage of fat tissue, muscle mass, and hormonal state), we only enrolled males in this study. This choice was guided by the higher percentage of muscle mass in males, which enabled us to detect subtle differences in a relatively small sample in energy consumption related to fat‐free mass (FFM), which consists largely of muscle tissue. The patients with PD were not on dopaminergic therapy (levodopa or agonists). Two of the PD patients were on symptomatic therapy (amantadine), and 6 were taking MAO‐B inhibitors.

The participants were assessed on the Motor Unified Parkinson's Disease Rating Scale (UPDRS) on the day of the test. The level of regular physical activity was evaluated using the Physical Activity Scale for the Elderly^6^ and was similar between groups (t test *P* > .11 for all questions; see Table S1). To exclude unrelated factors that can alter baseline metabolism, participants were instructed to avoid eating (4 h), drinking caffeinated beverages (2 h), and smoking (2 h) before the procedure.^7^

### 2.2 Measurement of FFM and oxygen consumption

The FFM measurement was calculated for each patient by bioelectrical impedance analysis using a body composition monitor (Tanita model BC‐545, Tanita Corporation, Tokyo, Japan). The body composition monitor was validated against dual‐energy X‐ray absorptiometry^8^ prior to the test. Indirect calorimetry was performed by measuring V̇O_2_ (oxygen consumption) using a metabolic cart with a built‐in spiroergometry (Zan 600, nSpire Health GmbH, Oberthulba, Germany), which measures breath‐by‐breath oxygen consumption and carbon dioxide output.

### 2.3 Spirometry procedures

To assess resting energy expenditure, participants sat in a comfortable armchair, with their torso leaning back at 50 to 70 degrees, for 10 minutes. All respired air was collected by a well‐fitted face mask. Calculations were based on the measurements obtained at minutes 8 to 10 (steady state). Average values of V̇O_2_ in liters per minute were multiplied by 4.9 to calculate caloric consumption in kilocalorie per minute and again by 1440 (number of min per 24 h) to calculate daily resting energy expenditure. We then measured V̇O_2_ in 4 sequential conditions—3 minutes of relaxed sitting on the bike, 4 minutes of unloaded cycling (no resistance, constant cycling speed 60 rpm), and 6 minutes of 40‐W loaded cycling (constant cycling speed 60 rpm). This was followed by a 4‐minute recovery period in which participants stopped cycling but continued to sit relaxed on the bike. The working load was well below the anaerobic threshold of all participants and ensured steady‐state conditions. The respiratory exchange ratio was similar between groups and did not exceed 0.9. During exercise, the patients' blood pressure, oxygen saturation, and electrocardiography were monitored. Visual inspection of the V̇O_2_ graphs revealed that during these time intervals, oxygen consumption was stable.

### 2.4 Statistics

Our experiment (10 individuals in each arm) was designed to detect at least a 20% increment of energy expenditure in the PD group (1‐sided test with *P* < .1), assuming intra‐group standard deviation of 20% of the group's mean with a power of 80%.

## 3 RESULTS

We measured the energy expenditure of 10 male patients with early PD and compared their results against 10 matched individuals without PD (participant characteristics are summarized in Table 1). Patients with PD reported a relative recent onset of symptoms (within 1‐5 y). The UPDRS motor scores ranged from 14 to 33, indicating mild to moderate severity of disease. None of the patients used levodopa or dopamine agonists.

**Table 1:** Participant characteristics

Characteristic	PD (Mean ± SD)	Control (Mean ± SD)	*P‐*value
Age, y	60.5 ± 11.1	60.8 ± 10.4	.95
Weight, kg	77.4 ± 14.7	79.7 ± 12.0	.69
Height, cm	173.0 ± 6.4	173.9 ± 7.3	.81
Body fat, %	22.4 ± 6.3	24.7 ± 6.6	.46
Fat‐free mass, kg	56.8 ± 9.6	56.6 ± 6.5	.96
BMI	25.9 ± 4.9	26.5 ± 4.7	.80
Duration of symptoms, y	2.60 ± 1.4	NA	NA
UPDRS part III scoring	20.6 ± 6.48	NA	NA
Use of PD medications (#): MAO‐B inhibitor Amantadine	6/10 2/10	NA	NA

**Figure 1:**
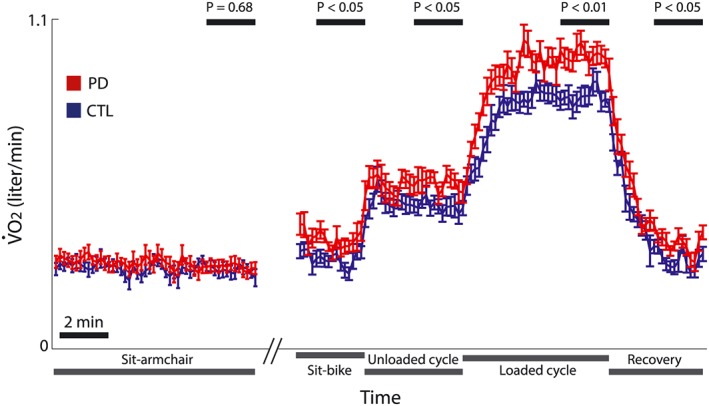
Breath‐by‐breath measure of oxygen consumption at rest and during exercise. Average rate of oxygen consumption (V̇O_2_) was calculated in patients with Parkinson disease (PD, red) and in healthy controls (CTL, blue) at a 10‐s sampling interval. Vertical bars represent standard error of the mean. The conditions are marked below the x‐axis, and the time intervals used for inter‐group comparison are marked above by black bars (average of the last 2 min for each condition). A time scale of 2 min is depicted by the black bar

The resting energy expenditure, calculated during supported sitting from measured V̇O_2_, was similar between groups (Figure 1 and Table 2). Energy expenditure was calculated as 1930 ± 390 kcal/day in PD and 1869 ± 310 kcal/day in controls (*P* = .68, t test). In contrast to the resting state with a supported body trunk posture, V̇O_2_ values in all the other conditions were significantly higher in the PD than in the control group (Figure 1 and Table 2).

**Table 2:** Oxygen consumption (V̇O_2_) in the different conditions

	V̇O_2_ (Mean ± SD in L/min)		
Condition of measurement	PD	Control	*P‐*value
Sitting in armchair	0.27 ± 0.06	0.27 ± 0.04	.68
Sitting on bicycle	0.36 ± 0.06	0.29 ± 0.05	.02
Unloaded cycling	0.56 ± 0.07	0.48 ± 0.07	.03
Loaded cycling	0.98 ± 0.09	0.84 ± 0.09	.003
Recovery	0.35 ± 0.06	0.29 ± 0.05	.048

To rule out the possibility of a confounding contribution of differences in muscle mass between individuals, we adjusted the V̇O_2_ to measure FFM (in liters per min per kilogram). This analysis yielded the same results, namely, a similar rest V̇O_2_ per kilogram in the PD and control groups (*P‐*value = .53) and a significantly higher V̇O_2_ per kilogram in the PD than in the controls in all 4 other conditions (*P*‐values = .005, .03, .04, and .045, respectively).

The physical tasks were increasingly demanding. When sitting in the armchair, both the axial and limb muscles were largely inactivated. Unsupported bike sitting required the activation of axial muscles, and for patients within each group, V̇O_2_ values were significantly higher while sitting on bicycle seat than when sitting in the armchair (PD: *P* = .0001, CRL: *P* = .01, paired t test). Unloaded cycling also required the activation of lower limb muscles. This activation further increased with loaded cycling. This served to test the hypothesis that the addition of muscle effort at different intensity levels would increase the abnormally high V̇O_2_ in PD. To calculate the additional oxygen consumption related to each of the exercises, for each individual, we subtracted his mean V̇O_2_ value at rest (calculated over the last 2 min of armchair rest) from the measured mean V̇O_2_ in each of the conditions. The increment in V̇O_2_ consumption in each phase was steeper in PD than in controls but was not statistically significant: The addition of unloaded cycling led to a V̇O_2_ increment of 0.20 ± 0.03 L/min in PD and 0.19 ± 0.06 in the controls (*P* = .53). The addition of a constant work load further increased V̇O_2_ 0.42 ± 0.08 L/min in PD vs only 0.36 ± 0.08 in controls (*P* = .09).

There were no correlations between the motor severity of the disease (total UPDRS III) and the increment of V̇O_2_ relative to rest in any of the conditions (for bike rest, unloaded, and loaded cycling, *P* = .98, .46, and .86, respectively). Correlating the different subcomponents of the UPDRS III (rigidity, tremor, bradykinesia, and axial) with the increment of V̇O_2_ relative to rest did not yield any significant results (all *P*‐values > .08), except for a significant correlation between tremor and increased energy expenditure during unsupported bike sit (*R* = .69, *P* = .03). This single significant correlation, among multiple statistical tests, is of uncertain clinical significance.

## 4 DISCUSSION

By measuring oxygen consumption, we showed that in a homogenous population of males with early PD who were not on dopaminergic therapy, the energy expenditure at rest did not increase. Physical activity, on the other hand, led to an increment in energy expenditure that was higher than in the controls. Evidence for abnormally increased energy expenditure emerged from the recruitment of axial muscles to support the body trunk in a sitting posture.

Our observation of normal resting energy expenditure in PD is consistent with previous studies^2^, ^5^ but conflicts with others.^3^, ^4^ These contradictory results may have to do with unmatched PD and control group gender,^3^ a lack of adjustment for FFM,^4^ or the recruitment of patients on medication that modulates metabolic rate such as beta‐blockers or thyroid hormone replacement therapy or individuals who had undergone deep brain stimulation.^9^ We avoided these issues by only testing male patients (for their higher proportion of FFM), matching for anthropomorphic parameters, and excluding patients on potentially confounding medication or post‐PD surgeries.

Daily energy expenditure, which sums both resting energy expenditure and the energy consumption induced by physical activity, was found to be lower in PD than in the non‐affected controls when was measured over several days by a doubly labeled water technique.^5^ In light of the normal resting energy expenditure, the differences between the 2 groups thus resulted from differences in daily activity. Activity is prone to change with disease progression, motivation to exercise, etc. We did not measure daily energy expenditure but rather showed that similar physical activity is more energy consuming in PD compared to unaffected participants. We cannot rule out the possibility that impaired movement coordination in PD patients during cycling contributed to increased energy consumption in this group. However, our observation that the maintenance of trunk posture also requires increased energetic cost in PD rules out this possibility as a sole explanation. This last observation has not been documented in previous studies.

The energy consumed by an active muscle for a given work load reflects the muscle's energetic efficiency. We showed that in PD, muscles may have lower energetic efficiency. Muscle energy consumption is governed to a great extent by the number, size, and concentration of mitochondria in muscle tissue. It remains unclear whether there is impaired mitochondrial function in the muscles of individuals with PD.^10^, ^11^, ^12^ Our work lends weight to the possibility that mitochondrial respiratory chain enzymes have decreased activity in PD.^12^ Another possibility is that the energetic efficacy in PD is normal but that muscle rigidity creates an additional load for the active muscle. In this study, V̇O_2_ was not correlated with the severity of PD symptoms (except for a positive correlation with tremor severity during unsupported bike sit, which is of uncertain clinical significance). This absence of correlation, however, might stem from the small sample size and the relatively narrow range of disease severity.

Weight loss in PD is multifactorial.^13^ Caloric disequilibrium is not only due to increased energy expenditure but also due to reduced caloric intake secondary to depression, hyposmia, and dysphagia. Hormones such as leptin may also play a role in weight loss in PD.^14^ We did not asses caloric intake in this study or follow the weight of participants longitudinally. We can, therefore, only speculate that the increased energy expenditure, which is limited to the active state, could be considered a contributing factor to weight loss observed in PD patients in the early phases of the disease.

## 5 PERSPECTIVES

It has been hypothesized that weight loss, a major contributor to impaired quality of life in PD,^15^ is the result of increased resting energy expenditure, yet conclusive evidence for this hypothesis has been lacking. This study demonstrated that increased energy expenditure in PD patients occurs only during posture maintenance and physical activity, not during rest. Recent research showed that training with Nordic walking poles improved motor function and locomotion in PD patients.^16^ Nordic walking poles increase stability of gait during walking and may off‐load posture‐maintaining accessory muscles that, as we shown, are overworked in these patients. Based on our results, we predict improvement in exercise energetics for PD patients in conjunction with muscular function improvement following this type of training.

## CONFLICT OF INTEREST

The authors declare no conflicts of interest.

## Supporting information

Table S1. Physical Activity Scale for the Elderly (PASE)
