# Supplementary material for: Increased energy expenditure during posture maintenance and exercise in early Parkinson disease
Source: Health Sci Rep. 2017 Aug 22;1(1):e14. doi: 10.1002/hsr2.14 (PMC6266470; doi:10.1002/hsr2.14)
Supplement: Supplementary file 1 — Table S1. Physical Activity Scale for the Elderly (PASE) [file HSR2-1-e14-s001.docx]

**Supplementary Table 1. Physical Activity Scale for the Elderly (PASE)**

| **Activity** | **PD**  **(Mean ± SD)** | **Control**  **(Mean ± SD)** | **P Value** |
| --- | --- | --- | --- |
| 1. Reported daily sitting (minutes) | 471 ± 205 | 426 ± 148 | 0.62 |
| 2. Reported daily walking (minutes) | 42 ± 37 | 75 ± 40 | 0.12 |
| 3. Reported daily light sport (minutes) | 8 ± 13 | 3 ± 9 | 0.18 |
| 4. Reported daily moderate sport (minutes) | 10 ± 13 | 25 ± 36 | 0.21 |
| 5. Reported daily strenuous sport (minutes) | 18 ± 24 | 8 ± 14 | 0.27 |
| 6. Reported daily endurance exercise (minutes) | 4.2 ± 6 | 1 ± 3 | 0.20 |
| 7. Reported daily light housework (minutes) | 27 ± 39 | 18 ± 18 | 0.43 |
| 8. Reported daily moderate housework (minutes) | 5 ± 9 | 12 ± 18 | 0.26 |
| 9. Reported daily heavy housework (minutes) | 48 ± 121 | 3 ± 9 | 0.28 |
